# Supplementary figures and images for: Aberrant light sensing and motility in the green alga Chlamydomonas priscuii from the ice-covered Antarctic Lake Bonney
Source: Plant Signal Behav. 2023 Mar 8;18(1):2184588. doi: 10.1080/15592324.2023.2184588 (PMC10012900; doi:10.1080/15592324.2023.2184588)

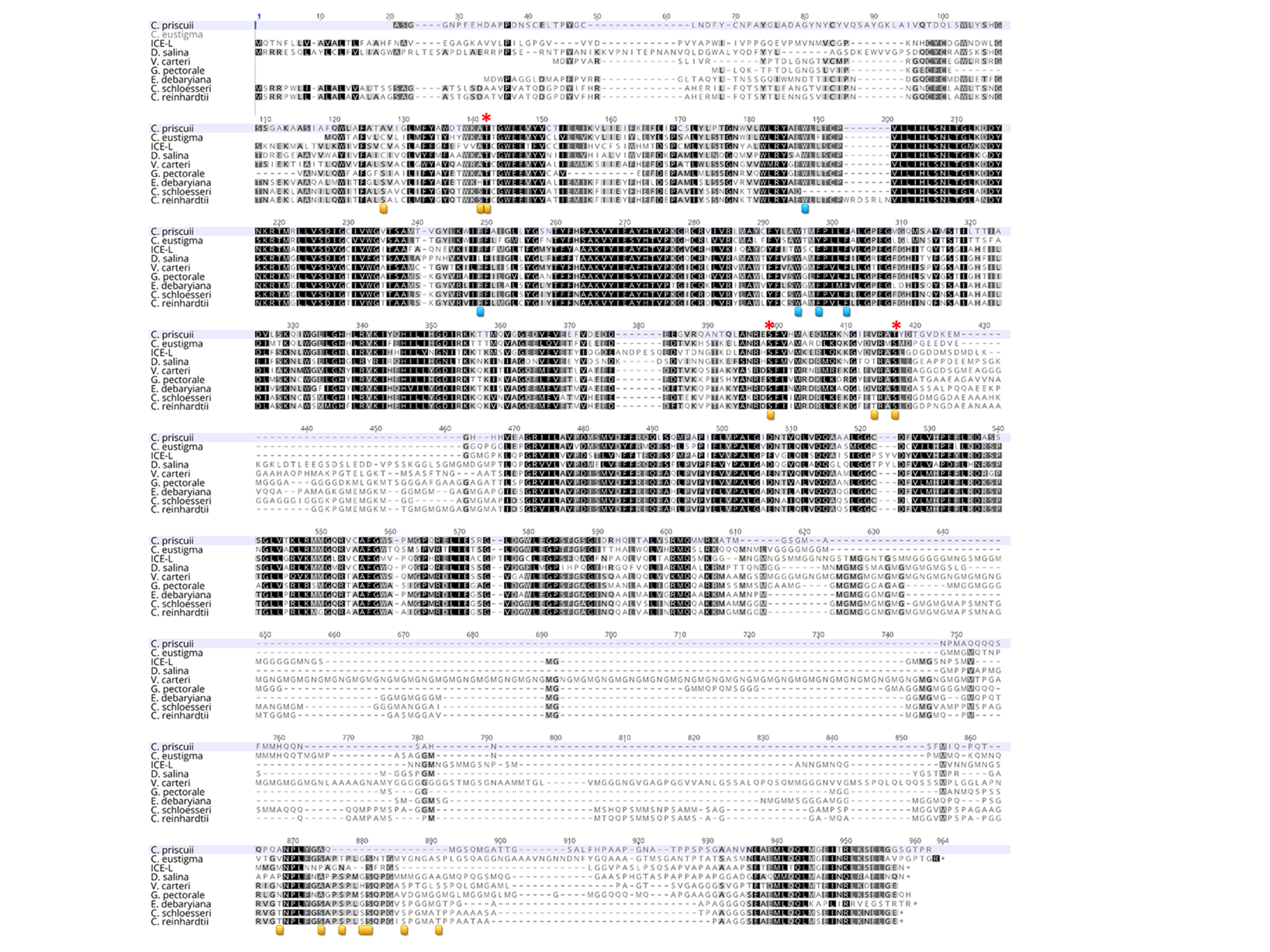

Supplement: Supplemental Material [file KPSB_A_2184588_SM5346.zip › Supplementary_Figure_1.TIF]

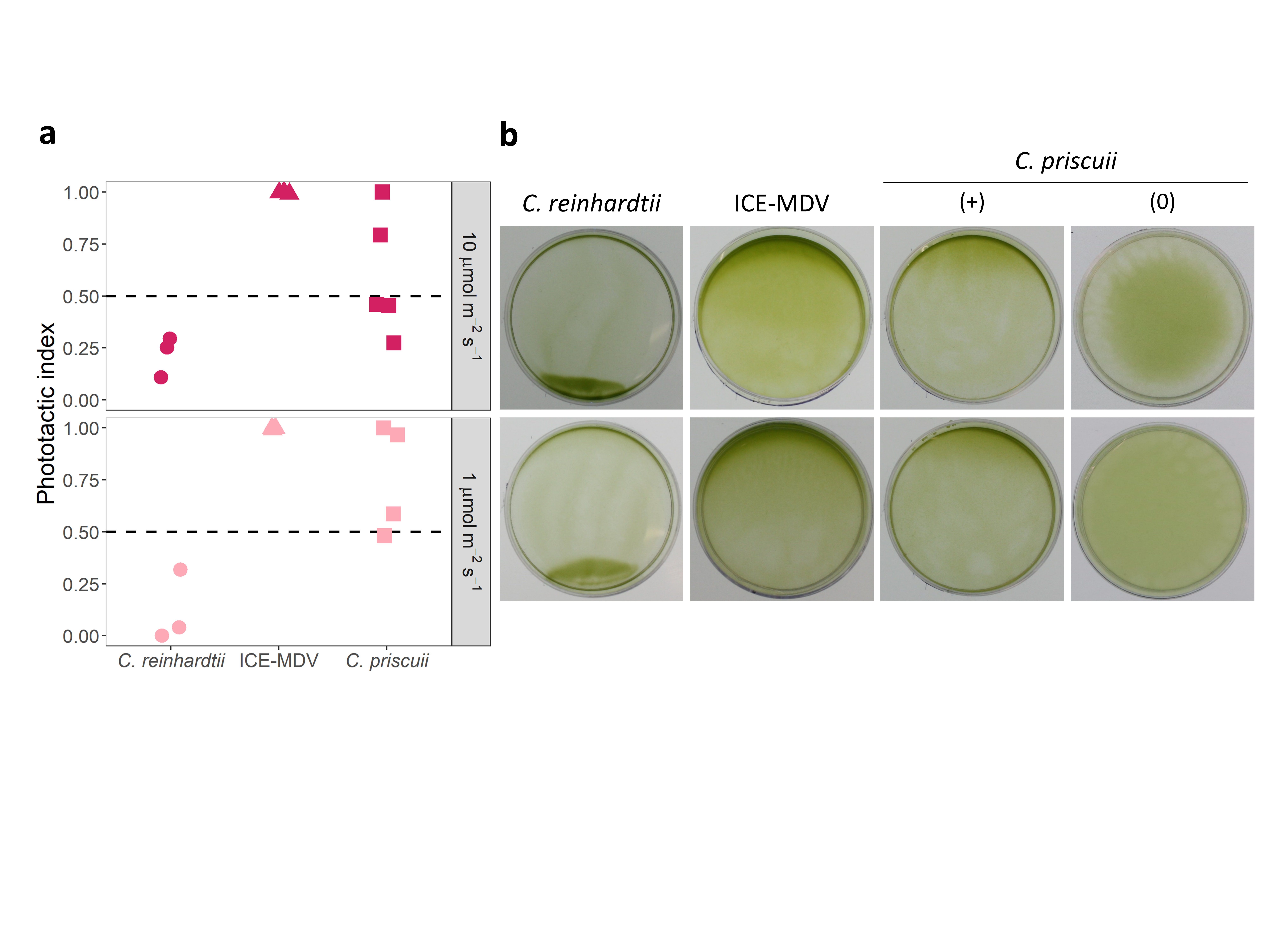

Supplement: Supplemental Material [file KPSB_A_2184588_SM5346.zip › Supplementary_Figure_2.TIF]

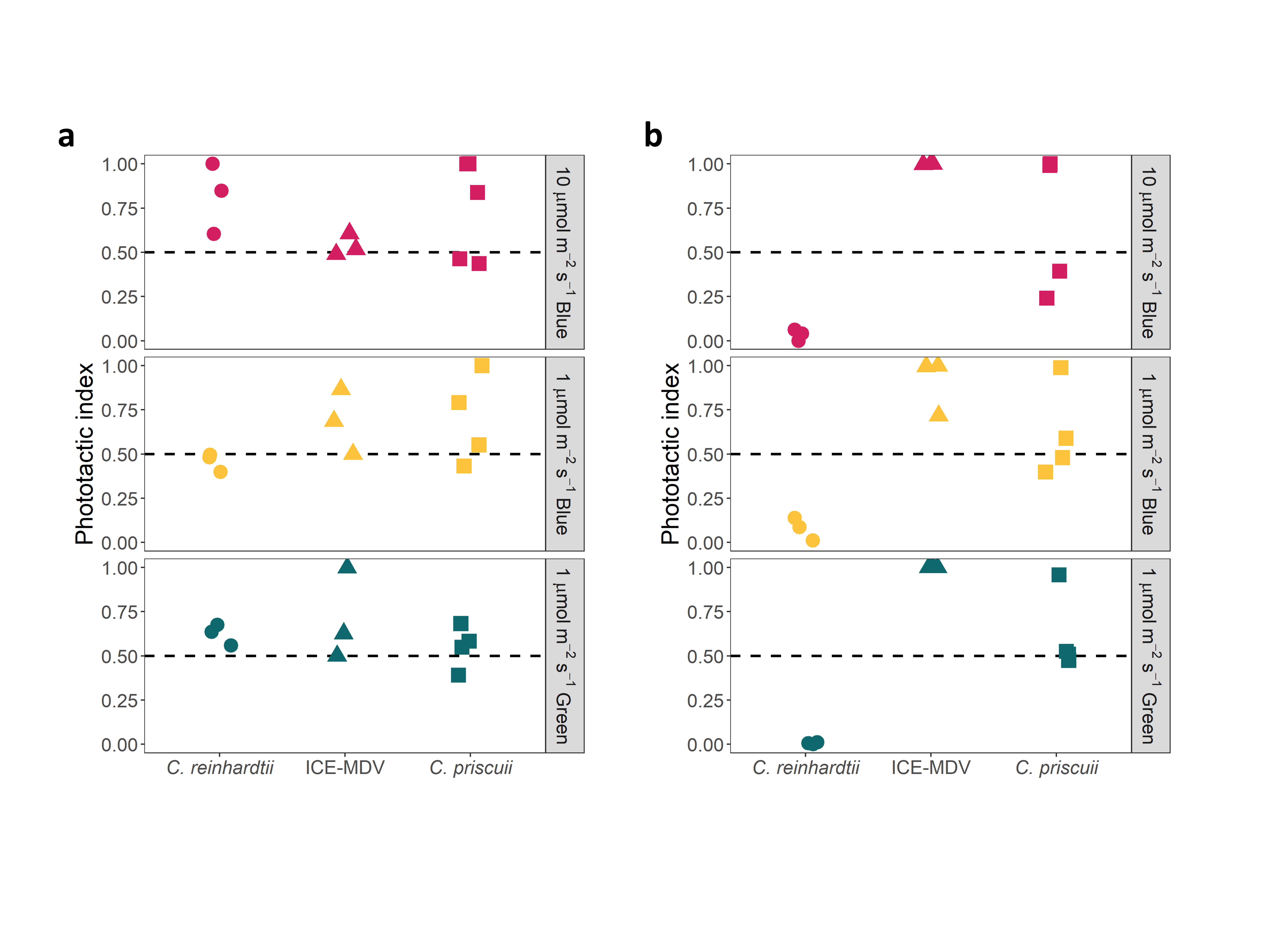

Supplement: Supplemental Material [file KPSB_A_2184588_SM5346.zip › Supplementary_Figure_3.TIF]

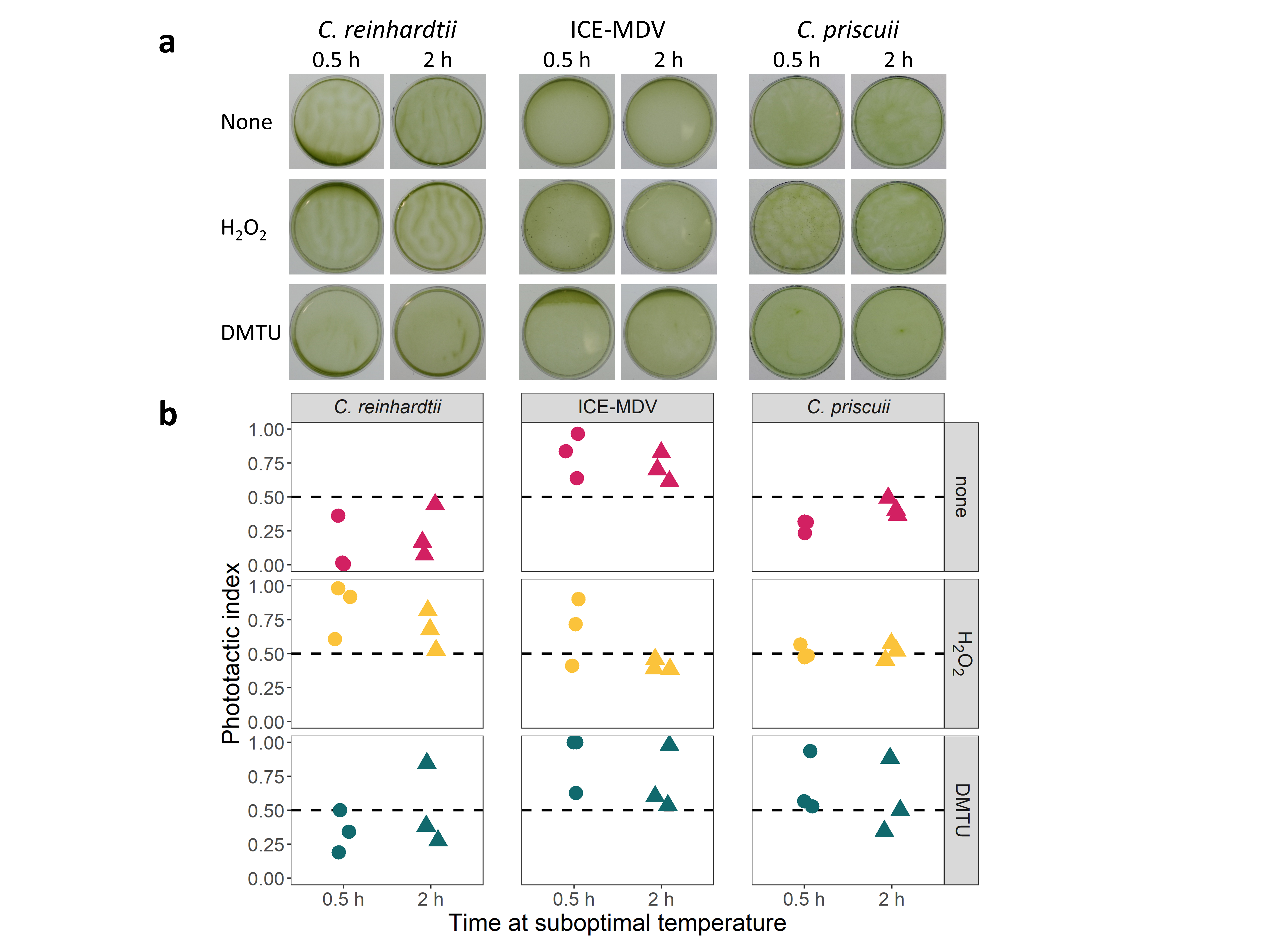

Supplement: Supplemental Material [file KPSB_A_2184588_SM5346.zip › Supplementary_Figure_4.TIF]

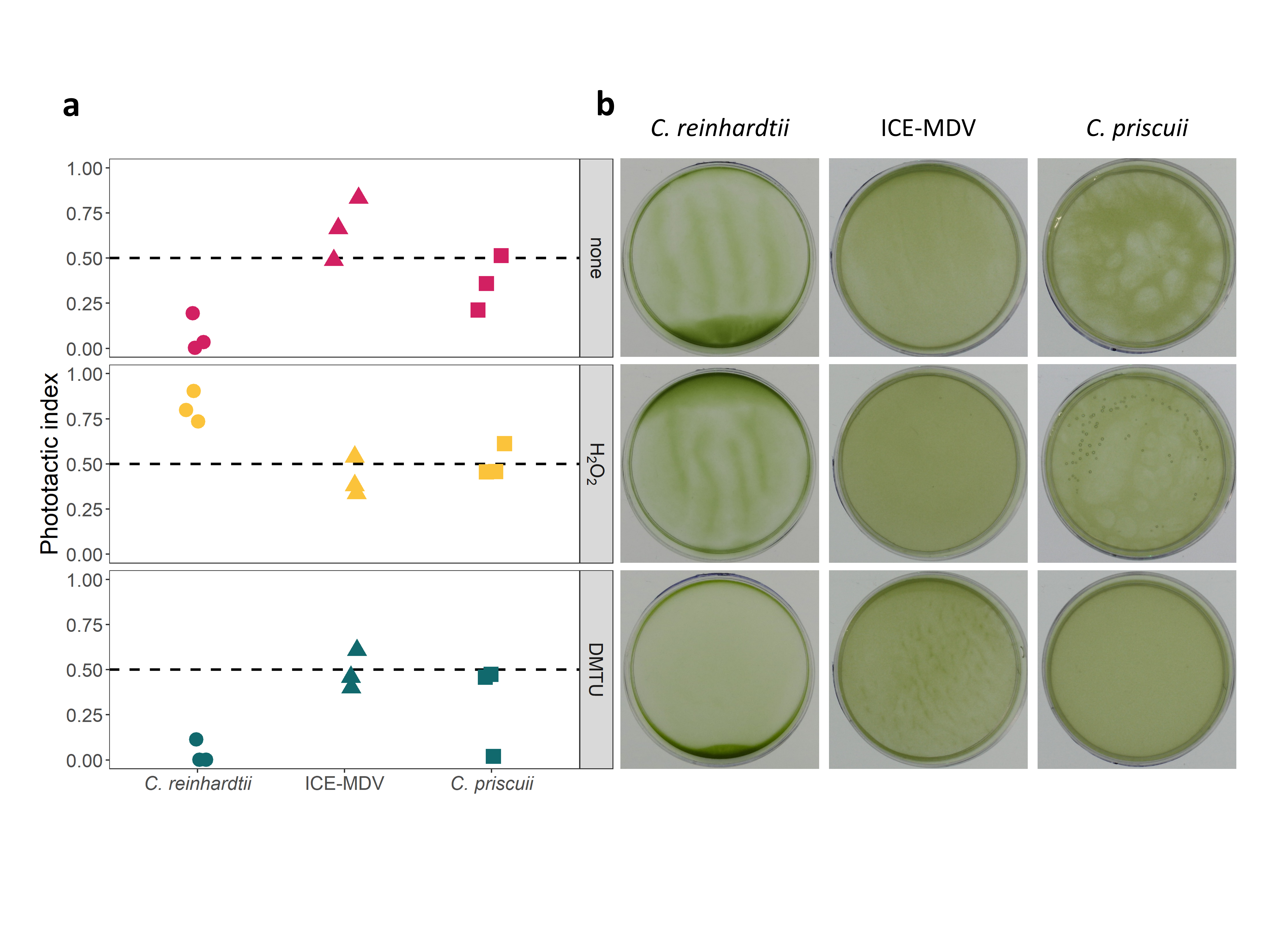

Supplement: Supplemental Material [file KPSB_A_2184588_SM5346.zip › Supplementary_Figure_5.TIF]
